# Socioeconomic disparities in physical activity among Swedish women and trends over time – the population study of women in Gothenburg

**DOI:** 10.1080/02813432.2018.1499599

**Published:** 2018-11-05

**Authors:** Maria Waller, Lauren Lissner, Dominique Hange, Valter Sund, Ann Blomstrand, Cecilia Björkelund

**Affiliations:** a Department of Public Health and Community Medicine/Primary Health Care, Institute of Medicine, Sahlgrenska Academy, University of Gothenburg, Gothenburg, Sweden;; b Department of Public Health and Community Medicine/Section for Epidemiology and Social Medicine, Institute of Medicine, Sahlgrenska Academy, University of Gothenburg, Gothenburg, Sweden

**Keywords:** Physical activity, socioeconomy, longitudinal study, cohort comparison, women, trends over time

## Abstract

**Objective:** To explore secular trends in physical activity in relation to socioeconomic position in middle-aged women, with focus on whether the social gaps have become wider, narrower, or remain unchanged.

**Design:** Cohort comparisons between two representative samples of women, recruited in 1980–81 and 2004–05 as a part of the Population Study of Women in Gothenburg.

**Setting:** Gothenburg, the second largest city of Sweden, with ≈ 450 000 inhabitants.

**Subjects:** Population-based cohorts of 38- and 50-year-old women, invited in 1980–81 and 2004–05 to free health examinations. The study population in 1980 was *n* = 477, 38- and 50-year-old women born in 1930 (*n* = 355) and 1942 (*n* = 122), and in 2004 *n* = 500, 38- and 50-year- old women born in 1966 (*n* = 207) and 1954 (*n* = 293).

**Main outcome measure:** Physical activity at work and leisure time. Socioeconomic position was defined based on socio-occupational group and level of education. Physical activity during work and leisure time was based on questionnaires.

**Results:** On average 38- and 50-year-old women were more physically active at work and leisure time in 2004–05 compared to 1980–81; odds ratio (OR) for increase over time for physical activity at work for 38-year-olds: 2.59, (95% confidence interval (CI) 1.65–4.07), and for 50-year-olds: OR 2.09 (1.52–2.88); OR for increase physical activity leisure time in 38-year-olds: 1.93 (1.25–2.98), and in 50-year-olds 2.04 (1.49–2.79). There were no significant differences between socioeconomic groups in physical activity levels changes over time.

**Conclusion:** Women in different socioeconomic groups improved their physical activity at work and leisure time to the same extent from 1980 to 2004, indicating that the socioeconomic gap in physical activity is neither increasing nor decreasing.

**Key Points**
  The gap in physical activity levels between socioeconomic groups seems to have remained stable for middle-aged women the last 25 years.
  • However, women were more physically active in 2004 at work and during leisure time, independent of socioeconomic position, compared to 1980.
  • It remains a great challenge to create structures that enable these behaviours for all social groups.

## Introduction

Physical inactivity is the fourth leading risk factor for mortality [1], and in particular, cardiovascular disease, diabetes, and cancer are strongly linked to physical inactivity. One third of adults and the majority of adolescents do not reach the levels of physical activity recommended in public health guidelines [2]. Strong associations have been observed between leisure time physical activity and well-being in women [3]. Physical activity on prescription is shown to improve health [4]. Trend data from high-income countries show that occupational physical activity is decreasing, but leisure time physical activity has increased in adults [2]. The levels of leisure time physical activity in several Nordic countries have also shown signs of increasing between 1980 and 2005 [5,6]. In Finland, the prevalence of high occupational physical activity has decreased during the last 30 years [6]. In Sweden, a positive trend has been observed concerning leisure time physical activity [7–9]. On the other hand, the Eurobarometer from 2014 [10], suggested that Sweden was one of the countries with the lowest daily physical activity in Europe.

Major improvements have occurred in the last thirty years in terms of women's health development. At the same time, increased educational opportunities and changing economic resources have affected the employment possibilities for middle-aged women, resulting in changes in their full-time employment rates, from around 35% in the 1970s to around 55% in 2004 [11].

In the Prospective Population Study of Women in Gothenburg, four population-based samples of middle-aged women (38- and 50-year-olds) participated in physical examinations with questionnaires on lifestyle, in order to document secular trends in cardiovascular health indicators [12]. The representative samples of 38- and 50-year-olds described their usual physical activity habits with the same questionnaires in 1968–69, 1980–81, 1992–93, and 2004–05, respectively [12–15]. As reported previously [15], mean leisure time physical activity was found to be significantly higher in later born cohorts; in 1980, around 24% were physically active compared with 40% in 2004. Several cardiovascular risk factors related to lifestyle were improved in middle-aged women in the latest decades. Most of the positive trends were observed in women with both low and high physical activity [15]. Reduction of risk factors was apparent in women with a high as well as low level of physical activity. Smoking declined most in women with high levels of physical activity [15].

These trends concerning physical activity in middle-aged women have also been observed in other populations [7,8]. However, the relationship between socioeconomic position and changes in physical activity levels has been studied less frequently, and it is not known whether the trends are uniform in different socioeconomic groups of women. Thus, the purpose of this study was to explore whether the gap between middle-aged women’s physical activity levels in different socioeconomic groups is widening or narrowing in Sweden.

## Aim

The specific aim was to examine differences in physical activity levels (at work and leisure time) between different socioeconomic groups in the population of 38- and 50-year old women in 1980–81 and 2004–05, respectively, and to explore whether the group differences have become wider, narrower, or unchanged.

## Material and methods

### The Prospective Population Study of Women in Gothenburg

**Figure 1.**
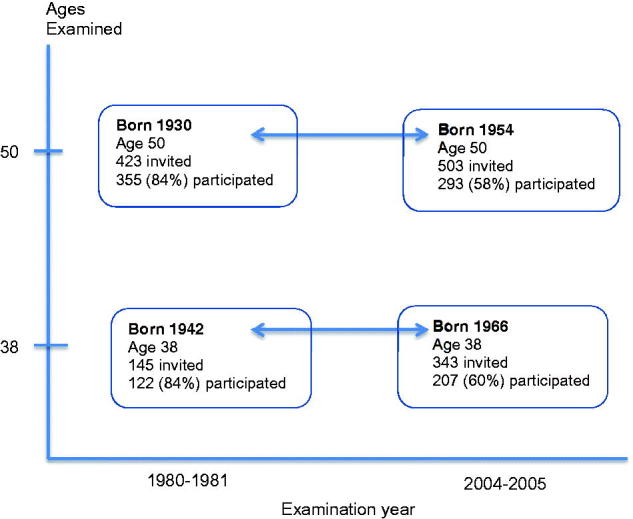
Description of the four 38- and 50-year-old age groups of women examined in the Population Study of Women examinations in 1980–81 and 2004–05, respectively. Year of birth, number of invited women and number of women who participated in the respective examinations.

From the Population Study of Women in Gothenburg [14,15], ongoing from 1968 to 69, we retrieved data on population-based randomised samples of 38- and 50-year-old women who were examined in 1980–81 and in 2004–05, respectively (Figure 1). The study population in 1980 was *n* = 477, 38-year-old women were born in 1942 (*n* = 122), and the 50-year-old women were born in 1930 (*n* = 355). The study population in 2004 was *n* = 500, the 38-year-old women were born in 1966 (*n* = 207), and the 50-year-old women were born in 1954 (*n* = 293).

Due to major changes in the Swedish educational system and women’s employment patterns between the 1960s and 1980s, it was not considered meaningful to include earlier-born cohorts that had also been investigated at these ages (cited original reference [12] to baseline cohort).

The total participation rate was 84% (*n* = 477) in 1980–81 and 59% (*n* = 500) in 2004–05 (Figure 1). In the current study we examined physical activity at work and during leisure time in relation to socio-occupational group and level of education. The women represent two different generations of middle-aged women, all of whom have undergone examinations of physical and mental health status and answered questions on physical activity at work and during leisure time.

Due to the decreasing participation rate in the 2004–05 examination, a register-based comparison was made between participants and non-participants [15]. Non-participants were more likely to belong to a lower socioeconomic group, as observed in most population studies. The participants had higher income and were more likely to have been born in Sweden. However, there were no differences in hospital admission rate, marital status, or place of residence between participants and non-participants.

### Physical activity indicator

A questionnaire was developed to describe physical activity at work and during leisure time in population studies in Gothenburg [13–15], based on the method described by Saltin and Grimby [16]. The questionnaire has been validated and found to discriminate activity levels adequately as compared with maximal oxygen uptake [17]. In all the examinations, women were interviewed by a physician and classified by this method into physical activity groups according to the extent of their activity, as follows. Four groups were classified with respect to physical activity – leisure time: (I) low physical activity, being almost totally inactive; (II) intermediate, indicating some physical activity for at least 4 h per week, e.g. walking or bicycling. (III) high, meaning regular physical activity e.g. gymnastics, gardening, tennis or golf and (IV) very high, for regular hard physical activity and competition, e.g. running or swimming several times a week.

Concerning physical activity at work, the women were again classified into groups according to the extent of physical activity. Women with light office work and no domestic work were assigned to group I. Group II included shop work, light industrial work or domestic work including the care of one child. Group III included for example, hospital work or domestic work including the care of two or more children. Group IV included heavy work together with domestic work, or just domestic work including the care of two or more children. This definition of work activity is unusual because domestic work is treated as an occupation [18]. This was adequate as the Population Study of Women in Gothenburg concerned midlife women, with changing occupation frequency in the different generations of women included in the study. Consequently, in case of unemployment, or long-term sickness absence/sickness pension, the corresponding actual physical activity level during daytime was registered accordingly and classified as physical activity at work.

### Socioeconomic position

Socioeconomic position was defined in terms of the social and economic factors that influence the positions that participants hold within the structure of society [19]. For the present study we used socio-occupational group and level of education to define socioeconomic position, as described below.

### Socio-occupational group

The women reported their occupations, and this information was used to categorise them into high, medium, and low occupational groups, according to Carlson´s standard occupations grouping system [20]. The group categorisation was carried out in accordance with the Swedish socioeconomic index [21], a widely accepted socio-occupational classification method that includes the number of years that a person has worked in an occupation in combination with an individual´s educational level [22].

### Educational group

In addition to the Swedish socioeconomic index, which is partly based on educational background, a specific educational grouping was defined. Years of education were reported by the women and converted into a 2-level ordinal variable: low and high educational attainment. In 1980–81, the category low education included all participants with primary school education or less (<7 years of school), which was compulsory at that time. In contrast to 1980–81, the category low education in 2004–05 comprised all participants who had completed 12 years of education.

### Statistics

The association between low physical activity at work/leisure time and low/high socio-occupational group/education was tested cross-sectionally at both time-points (Pearson Chi-Square test) and reported as odds ratios (Table 2(a)). The association between high physical activity at work/leisure time and low/high socio-occupational group/education was also tested cross-sectionally (Table 2(b)). Physical activity levels were dichotomised as follows: low (I) versus (II, III, IV) (Table 2(a)) and high (III, IV) versus (I, II) (Table 2(b)).

Ordinal logistic regression analyses were performed to compute odds for improvement between 1980–81 and 2004–05, in terms of odds ratios (OR) with 95% confidence intervals (CI) (Table 3). The dependent variables were four levels of physical activity (I–IV). Differences in physical activity between the two 38-year-old samples and the two 50-year-old samples were tested, adjusted separately for socio-occupational group and educational group. Differences were considered statistically significant at *p* < 0.05. Test of interactions between time and the socioeconomic position indicators showed no statistically significant differences.

## Results

### Descriptive data

**Figure 2.**
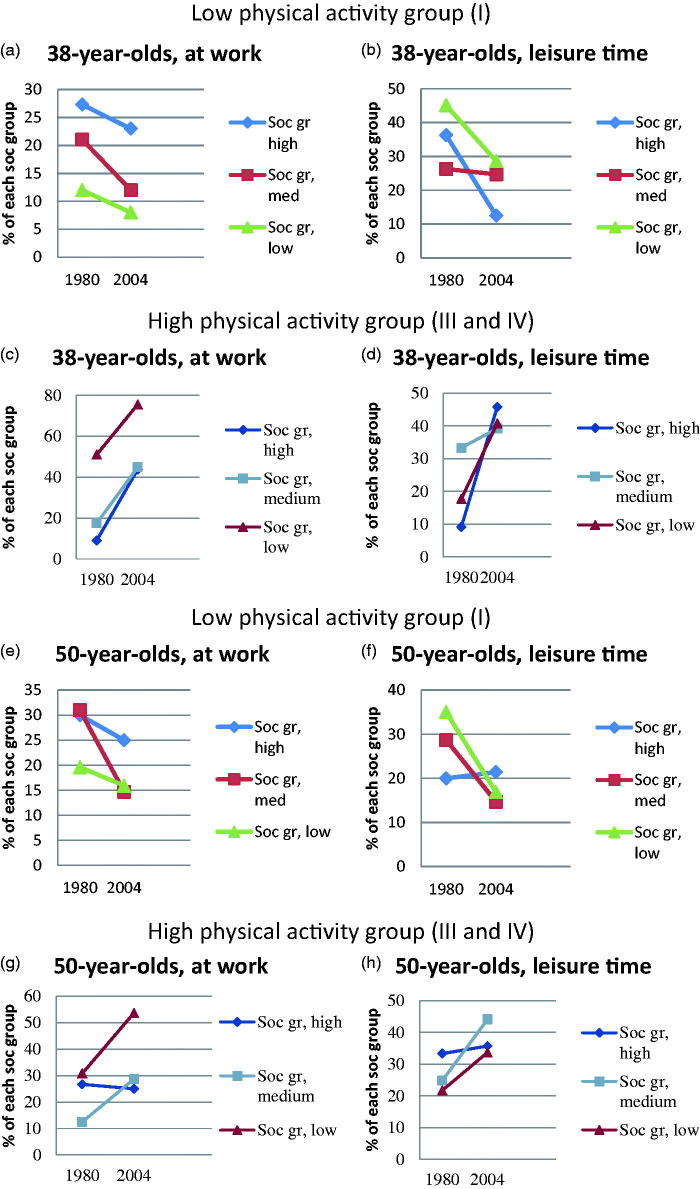
Change in the proportion of women who belonged to low/high physical activity group at work and leisure time, from 1980–81 to 2004–05 for socio-occupational group low, medium, and high, respectively, 38-year-olds (a-d) and 50-year-olds (e-h).

The two different generations of middle-aged women were stratified into two groups, those examined in 1980–81 and those in 2004–05. Table 1 presents descriptive data for 38- and 50-year-olds within each group, including number and frequencies, participation rates, marital status, smoking, employment status, physical activity at work and leisure time, socio-occupational level, and educational level. Figure 2 illustrates age-specific differences in physical activity at work and leisure time between 1980–81 and 2004–05. Concerning 38-year-olds, (a-d), physical activity at work and leisure time increased from 1980 to 2004 in all three socio-occupational groups. Concerning 50-year-olds, (e-h), physical activity at work and leisure time increased in two out of three socio-occupational groups. Both for 38- and 50-year-olds, there were no significant differences between physical activity levels in the different socio-occupational groups; all physical activity level changes were in the same direction and there was no increase in the gap between the different socio-occupational groups, but on the other hand, there was no decrease (Figure 2).

**Table 1.** Characteristics of 38- and 50-year-old women in the Population Study of Women in Gothenburg regarding the assessments performed in 1980–81 and 2004–05, respectively.

Variables/age	1980–81		2004–05	
	38 years	50 years	38 years	50 years
	*N* = 122	*N* = 355	*N* = 207	*N* = 293
	*n* (%)	*n* (%)	*n* (%)	*n* (%)
Marital status				
Married n (%)	79 (65)	261 (73)	90 (44)	154 (53)
Smoking	46 (38)	139 (39)	23 (11)	67 (23)
Employment status				
Full time = 35 h/w	48 (40)	112 (32)	126 (61)	179 (61)
Part time = 1-34 h/w	56 (46)	180 (51)	51 (25)	62 (21)
Unemployment	17 (14)	62 (18)	26 (13)	49 (17)
Physical activity – work				
I Low	22 (18)	96 (27)	26 (13)	50 (17)
II Intermediate	62 (51)	180 (51)	69 (34)	138 (48)
III High	33 (27)	69 (19)	97 (48)	93 (32)
IV Very high	5 (4)	10 (3)	11 (5)	6 (2)
Physical activity – leisure time				
I Low	42 (34)	114 (32)	46 (23)	48 (17)
II Intermediate	51 (42)	161 (45)	74 (36)	128 (44)
III High	28 (23)	76 (21)	72 (35)	104 (36)
IV Very high	1 (1)	4 (1)	12 (6)	9 (3)
Socio-occupational level				
Low	51 (43)	143 (47)	50 (25)	84 (29)
Medium	57 (48)	129 (43)	98 (50)	145 (51)
High	11 (9)	30 (10)	49 (25)	57 (20)
Educational level				
Low	43 (35)	228 (65)	89 (44)	143 (49)
High	79 (65)	127 (35)	114 (56)	150 (51)

### Physical activity level and association with socioeconomic position

#### Physical activity at work

When testing risk estimates for the association between different physical activity levels (outcome) and the socio-occupational level of a given sample (exposure), physical activity at work showed a significant negative association for 50-year-olds in 1980–81 between low physical activity (I vs. II, III, IV) and low socio-occupational level (Table 2(a)), OR 0.55, CI (0.32–0.93). A significant positive association was found between low physical activity and low education for 50-year-olds in 2004–05, OR 1.89 and CI (1.01–3.53).

**Table 2.** The association between: (a), low (I) vs. high (II, III, IV) physical activity (outcome) at work/leisure time, (b), high (III, IV) vs. low (I, II) physical activity (outcome) at work/leisure time, and low/high socio-occupational group and low/high education group, respectively, defined as the exposed group. Odds ratios (OR) with 95% confidence intervals (CI).(a) Low physical activity (I) versus (II, III and IV).

	Low physical activity – at work				Low physical activity – leisure time			
	1980–81		2004–05		1980–81		2004–05	
	OR (CI)	*p*	OR (CI)	*p*	OR (CI)	*p*	OR (CI)	*p*
Low socio-occupational Group								
38 years	0.47 (0.17−1.32)	0.15	0.49 (0.16−1.51)	0.21	2.12 (0.99−4.55)	0.053	1.53 (0.73−3.21)	0.26
50 years	0.55 (0.32−0.93)	**0.03**	0.88 (0.44−1.77)	0.73	1.45 (0.89−2.37)	0.14	1.02 (0.51−2.03)	0.95
Low education								
38 years	0.64 (0.23−1.78)	0.39	0.63 (0.27−1.49)	0.29	1.21 (0.56−2.63)	0.63	1.29 (0.66−2.51)	0.45
50 years	0.67 (0.41−1.08)	0.10	1.89 (1.01−3.53)	**0.045**	1.20 (0.74−1.93)	0.46	1.21 (0.64−2.26)	0.56
High socio-occupational Group								
38 years	2.13 (0.78–5.88)	0.15	2.04 (0.66−6.25)	0.21	0.47 (0.22−1.01)	0.053	0.65 (0.31−1.37)	0.26
50 years	1.82 (1.08–3.13)	**0.03**	1.14 (0.56−2.27)	0.73	0.69 (0.42−1.12)	0.14	0.98 (0.49−1.96)	0.95
High education								
38 years	1.56 (0.56–4.35)	0.39	1.59 (0.67−3.70)	0.29	0.83 (0.38−1.79)	0.63	0.78 (0.40−1.52)	0.45
50 years	1.49 (0.93–2.44)	0.10	0.53 (0.28−0.99)	**0.045**	0.83 (0.52−1.35)	0.46	0.83 (0.44−1.56)	0.56

**Table:** (b) High physical activity (III, IV) versus low (I, II).

	High physical activity – at work				High physical activity – leisure time			
	1980–81		2004–05		1980–81		2004–05	
	OR (CI)	*p*	OR (CI)	*p*	OR (CI)	*p*	OR (CI)	*p*
Low socio-occupational Group								
38 years	5.39 (2.31–12.58)	**<0.001**	3.85 (1.86–7.99)	**<0.001**	0.51 (0.21–1.25)	0.14	0.98 (0.51–1.89)	0.95
50 years	2.50 (1.43-4.38)	**<0.001**	3.03 (1.78–5.17)	**<0.001**	0.77 (0.45–1.31)	0.34	0.71 (0.42–1.22)	0.21
Low education								
38 years	1.81 (0.82–3.98)	0.14	1.72 (0.98–3.03)	0.06	0.63 (0.25–1.58)	0.32	1.45 (0.82–2.56)	0.20
50 years	2.30 (1.28–4.15)	**0.005**	1.31 (0.80–2.14)	0.28	0.94 (0.56–1.59)	0.83	0.74 (0.46–1.19)	0.21
High socio-occupational group								
38 years	0.19 (0.08–0.43)	**<0.001**	0.26 (0.13–0.54)	**<0.001**	1.96 (0.80–4.76)	0.14	1.02 (0.53–1.96)	0.95
50 years	0.40 (0.23–0.70)	**<0.001**	0.33 (0.19–0.56)	**<0.001**	1.30 (0.76–2.22)	0.34	1.41 (0.82–2.38)	0.21
High education								
38 years	0.55 (0.25–1.22)	0.14	0.58 (0.33–1.02)	0.06	1.59 (0.63–4.00)	0.32	0.69 (0.39–1.22)	0.20
50 years	0.43 (0.24–0.78)	**0.005**	0.76 (0.47–1.25)	0.28	1.06 (0.63–1.79)	0.83	1.35 (0.84–2.17)	0.21

Bold figures: Statistically significant differences between the cohorts.

Concerning high physical activity (VI, III vs. I, II) at work (Table 2(b)), statistically significant positive associations were found between high physical activity at work and low socio-occupational level in 38-and 50-year-olds, both in 1980–81 and in 2004–05. Concerning high physical activity at work and low education, a statistically significant positive association was found for 50-year-olds in 1980–81 (Table 2(b)).

#### Leisure time physical activity

No statistically significant associations were found between low leisure time physical activity and different socioeconomic groups (Table 2(a)). Similar findings were seen concerning high leisure time physical activity and different socioeconomic groups (Table 2(b)).

### Increase of mean physical activity level 1980–81 to 2004–05

#### Physical activity at work

Odds of increase of mean physical activity level at work for 38- and 50-year-olds over time (from 1980–81 to 2004–05) were statistically significant both when controlled for socio-occupational group as well as for level of education, respectively (Table 3). Odds for belonging to a higher level of physical activity were twice as high in 2004–05 compared to 1980–81. Test of interaction showed no interaction concerning socio-occupational group or level of education.

**Table 3.** Odds for increase over time (from 1980-81 to 2004-05) in mean physical activity level (using the four levels of the activity scale) at work and leisure time for 38- and 50-year-old cohorts, adjusted for socio-occupational group and educational level, respectively. Odds ratios (OR) with 95% confidence intervals (CI).

	Adjusted for Socio-occupational group1980–2004	Adjusted for Educational level 1980–2004
	OR (CI)	OR (CI)
Physical activity at work		
38-year-olds	**2.59** (1.65–4.07)	**2.29** (1.40–3.75)
50-year-olds	**2.09** (1.52–2.8`8)	**2.01** (1.35–2.98)
Physical activity leisure time		
38-year-olds	**1.93** (1.25–2.98)	**1.67** (1.04–2.70)
50-year-olds	**2.04** (1.49–2.79)	**2.27** (1.54–3.34)

Bold figures: Statistically significant differences between the cohorts in increase in mean physical activity levels in 1980 and 2004. There was no interaction between the effect of socio-occupational group and education.

The analysis of increase of mean physical activity level at work from 1980–81 to 2004–05 was repeated with only those women who were working (full employment or part time) and the results were in agreement with the results with home-working women included. Odds for belonging to a higher level of physical activity were then more than twice as high in 2004–05 compared to 1980–81; for 38-year-olds OR 2.72 (CI 1.69–4.38) and 2.36 (CI 1.41–3.96), and for 50-year-olds OR 3.06 (CI 2.15–4.35) and 3.09 (CI 2.00–4.77).

#### Leisure time physical activity

For 38- and 50-year-olds, the odds of increase over time (from 1980–81 to 2004–05) in mean leisure time physical activity level were statistically significant, both when controlled for socio-occupational group as well as for level of education, respectively (Table 3). Odds for belonging to a higher level of physical activity were around twice as high in 2004–05 compared to 1980–81. Test of interactions between socioeconomic position and time showed no interaction for either socio-occupational group or level of education.

## Discussion

Our results show that for 38- and 50-year-old women, mean physical activity level at work was higher in all socioeconomic groups in 2004 compared to 1980, a result that was confirmed also when excluding the group of home-working women. Concerning leisure time physical activity, 38- and 50-year-old women in all socioeconomic groups also showed higher mean physical activity level in 2004 compared to 1980. We found a positive association between high physical activity at work and low socioeconomic position, but no associations were found between leisure time physical activity and different socioeconomic groups.

The strengths of this study are that the uniformity of the examinations has been maintained over a long time period (24 years) as well as the long duration of the study. This is one of few cohort comparison studies of women´s physical activity level in relation to socioeconomic position within such a long term perspective. Another strength of this study is the complementary data both on physical activity at work and during leisure time. The standardised procedures and similar questionnaires used by the team of examining physicians enabled us to optimise comparability of physical activity levels as much as possible.

A limitation of the study is that the participation rate decreased over time. In the examination 2004–05, non-participants were more likely to belong to a lower socio-occupational group compared to participants [15]. It must be acknowledged that as participation decreased between examinations, the degree to which the participants were representative of middle-aged women in Gothenburg also decreased. However, the declining participation rate in our study, to around 60% in 2004–05, is comparable with other population surveys [23]. This can perhaps be explained by changes in societal gender roles; women seem to have less spare time [24] and perceive more mental stress [25]. As the non-participation rate was higher for the lower socioeconomic groups in 2004–05, a higher participation rate would on the other hand probably further strengthen the associations.

Another limitation is the fact that data were based on the women´s personal reports [26], as stated in questionnaires and physician interviews. On the other hand, the fact that the validated questionnaires used 1980–81 and 2004–05 were identical made comparison possible. However, it is acknowledged that objective methods for assessing physical activity levels are used in most contemporary surveys to supplement subjectively reported information [26].

In our study, mean physical activity level at work and during leisure time increased over time in all socioeconomic groups, as also has been observed in other studies, for example in Finland [27] and in Denmark [5]. Our results are mainly in agreement with an English study [28], where adults belonging to low socioeconomic groups were less likely to be active. An Australian study [29] showed that higher socioeconomic position was linked to higher total sitting and computer time, and lower TV viewing time.

It is encouraging that this increase in physical activity is seen in all socioeconomic groups, considering only 7% of middle-aged Swedish men and women managed to reach the criteria for national physical activity recommendations (150 min per week) [30]. Today's middle-aged women have seemingly come to recognise the importance of physical activity, and this appears to be the case regardless of socioeconomic group.These good health behaviours are likely to have a beneficial effect on health outcomes. Our results are not in agreement with other studies in Sweden, i.e. SALLS [7] and VIP [8], where the participants with higher education increased their physical activity -levels more than others. However, these studies are based only on data concerning leisure time physical activity. Possible reasons for the lack of a significantly widening gap shown in our study are perhaps the low numbers of participants and/or different definitions of physical activity and socioeconomic position variables.

Policymakers should work towards developing health-promoting structures in society especially involving groups with low socioeconomic position, but also engaging all inhabitants. Society and the health care system should work with methods such as “Pro-Health” [31,32], to facilitate and stimulate people to improve their lifestyle. We propose targeted efforts such as “Pro-Health”, a programme that has been shown to have an especially beneficial impact on vulnerable groups.

## Conclusions

In conclusion, women in western Sweden were more physically active in 2004–05 at work and leisure time independent of socioeconomic position, and the gap in physical activity levels between socioeconomic groups seems to have remained stable. How to create structures and environments that enable these behaviours for all socioeconomic groups remains a great challenge.

## Ethical approval

The Prospective Population Study of Women in Gothenburg and the Gerontological and Geriatric Population Studies in Gothenburg, Sweden were approved by the ethics committee of University of Gothenburg. The studies comply with the *Declaration of Helsinki* and informed consent has been obtained from the subjects.

## Disclosure statement

No potential conflict of interest was reported by the authors.
